# High-Coverage Serum Metabolomics Reveals Metabolic Pathway Dysregulation in Diabetic Retinopathy: A Propensity Score-Matched Study

**DOI:** 10.3389/fmolb.2022.822647

**Published:** 2022-03-17

**Authors:** Chengnan Guo, Depeng Jiang, Yixi Xu, Fang Peng, Shuzhen Zhao, Huihui Li, Dongzhen Jin, Xin Xu, Zhezheng Xia, Mingzhu Che, Mengyuan Lai, Ruogu Huang, Hui Wang, Chao Zheng, Guangyun Mao

**Affiliations:** ^1^ Division of Epidemiology and Health Statistics, Department of Preventive Medicine, School of Public Health and Management, Wenzhou Medical University, Wenzhou, China; ^2^ Center on Evidence-Based Medicine and Clinical Epidemiological Research, School of Public Health and Management, Wenzhou Medical University, Wenzhou, China; ^3^ Department of Community Health Sciences, College of Medicine, University of Manitoba, Winnipeg, MB, Canada; ^4^ Department of Nursing, School of Nursing, Wenzhou Medical University, Wenzhou, China; ^5^ The Second Affiliated Hospital of Zhejiang University School of Medicine, Hangzhou, China; ^6^ Center on Clinical Research, The Eye Hospital of Wenzhou Medical University, Wenzhou, China

**Keywords:** diabetic retinopathy, metabolomics study, metabolic pathway dysregulation, propensity score matching, integrated pathway analysis

## Abstract

**Background:** Diabetic retinopathy (DR) is a major diabetes-related disease linked to metabolism. However, the cognition of metabolic pathway alterations in DR remains scarce. We aimed to corroborate alterations of metabolic pathways identified in prior studies and investigate novel metabolic dysregulations that may lead to new prevention and treatment strategies for DR.

**Methods:** In this case-control study, we tested 613 serum metabolites in 69 pairs of type 2 diabetic patients (T2DM) with DR and propensity score-matched T2DM without DR *via* ultra-performance liquid chromatography-tandem mass spectrometry system. Metabolic pathway dysregulation in DR was thoroughly investigated by metabolic pathway analysis, chemical similarity enrichment analysis (ChemRICH), and integrated pathway analysis. The associations of ChemRICH-screened key metabolites with DR were further estimated with restricted cubic spline analyses.

**Results:** A total of 89 differentially expressed metabolites were identified by paired univariate analysis and partial least squares discriminant analysis. We corroborated biosynthesis of unsaturated fatty acids, glycine, serine and threonine metabolism, glutamate and cysteine-related pathways, and nucleotide-related pathways were significantly perturbed in DR, which were identified in prior studies. We also found some novel metabolic alterations associated with DR, including the disturbance of thiamine metabolism and tryptophan metabolism, decreased trehalose, and increased choline and indole derivatives in DR.

**Conclusions:** The results suggest that the metabolism disorder in DR can be better understood through integrating multiple biological knowledge databases. The progression of DR is associated with the disturbance of thiamine metabolism and tryptophan metabolism, decreased trehalose, and increased choline and indole derivatives.

## Introduction

Despite being possibly preventable and treatable, diabetic retinopathy (DR) is still the major microvascular complication of diabetes mellitus (DM) and the chief reason for vision impairment and blindness around the working-age population (Williams et al., 2019). World Health Organization (WHO) has considered DR to be prevented or treated as one of the principal eye conditions (Elmasry et al., 2019). From 1980 to 2018, the annual incidence and progression of DR have increased from 2.2 to 3.4%–12.7 and 12.3%, respectively (Sabanayagam et al., 2019). A recent meta-analysis reveals that DR is the only cause for the global growth of blindness in age-standardized prevalence between 1990 and 2020, especially in many parts of Asia and sub-Saharan Africa (Steinmetz et al., 2021). According to the Handan Eye Study, about 43.1% of people over 30 years old suffering from DM has been diagnosed as DR (Wang et al., 2009). Meanwhile, current treatment strategies, such as laser photocoagulation and anti-vascular endothelial growth factor (VEGF) injections, cannot always effectively control DR progression (Wang and Lo, 2018). So it is urgent to find new pathways associated with DR to achieve early prevention and treatment (Wang and Lo, 2018; Tomita et al., 2021).

Metabolome, which reflects the interplay of genetic and environmental factors, defines the information closest to the phenotype of the biological system under study (Chen et al., 2016; Ji et al., 2017). Since the functions of metabolites are not determined by epigenetic regulation or posttranslational modification like genes and proteins (Ji et al., 2017), metabolomics can uncover disease mechanisms that cannot be explored by other omics studies.

Previous blood metabolomics studies tried to find pathogenic pathways associated with DR using metabolic pathway analysis (Li et al., 2011; Chen et al., 2016; Liew et al., 2017; Rhee et al., 2018; Zhu et al., 2019; Xuan et al., 2020; Zuo et al., 2021). Based on plasma metabolomics, Li *et al* (Li et al., 2011) reported that lower level of *ω*-6 polyunsaturated fatty acids (PUFAs) is associated with proliferative diabetic retinopathy (PDR); Chen *et al* (Chen et al., 2016) demonstrated that pentose phosphate pathway is altered in moderate non-proliferative diabetic retinopathy (NPDR) patients after matching glycosylated hemoglobin (HbA1c); Rhee *et al* (Rhee et al., 2018) demonstrated glutamine and glutamic acid-related pathways is dysregulated in DR after matching age and sex; Zhu *et al* (Zhu et al., 2019) reported alanine, aspartate and glutamate metabolism, caffeine metabolism, beta-alanine metabolism, purine metabolism, cysteine and methionine metabolism, sulfur metabolism, sphingosine metabolism and arginine and proline metabolism are all enriched in PDR patients. Based on serum metabolomics, Xuan *et al* (Xuan et al., 2020) demonstrated that energy metabolism, amino acid metabolism, and lipid metabolism are disordered in DR patients after matching age and sex, and further emphasize the value of serum metabolomics studies for ascertaining its pathogenesis; Zuo *et al* (Zuo et al., 2021) demonstrated that linoleic acid metabolism, alanine, aspartate and glutamate metabolism and phenylalanine metabolism are enriched in DR patients after matching age, sex, body mass index (BMI) and HbA1c.

Though the above-mentioned blood metabolomics studies have been devoted to finding DR-related pathways, DR-related metabolomics studies are still in the early stage (Xuan et al., 2020). Most DR-related metabolic pathways identified in the above studies are restricted to those related to energy, amino acid, and lipid metabolism, which is mainly due to the application of threshold-based pathway analysis as well as the incomplete existing metabolic pathways map. Over-representation analysis is the most common applied threshold-based pathway analysis method in previous DR metabolomics studies, which overlooks the metabolites that individual effects are weak but coordinated changes in sets of functionally related metabolites have significant effects (Khatri et al., 2012). Furthermore, since there is no complete database of human metabolic pathways (Barupal et al., 2012), metabolic pathway analysis based on a single database may be not sufficient to fully understand the DR-perturbed metabolic pathways.

Herein, we comprehensively investigated DR-related serum metabolome changes in a propensity score matching (PSM)-designed case-control study using metabolic profiling data obtained from the ultra-performance liquid chromatography-tandem mass spectrometry (UPLC-MS/MS) platform. This study aimed to systematically evaluate metabolic pathway dysregulation during the development of DR using metabolic pathway analysis, chemical similarity enrichment analysis (ChemRICH), and integrated pathway analysis.

## Materials and Methods

### Study Population

This was a two-center, PSM-based case-control study. The rationale and study design of the study were reported previously. Briefly, from August 2017 to June 2018, we enrolled 950 volunteers, including 112 type 2 diabetic patients (T2DM) patients without DR and 83 with DR, and 755 health controls, aged over 35 ears from the endocrinology departments of affiliated hospitals of two medical universities in Wenzhou and Anhui provinces, China. All enrolled participants had no histories of the following diseases such as any other eye diseases, type 1 diabetes, cardiovascular disease, heart failure, cancer, infectious disease, or other chronic systemic diseases. The diagnosis of T2DM was applied strictly according to the standard criteria recommended by WHO since 1999. The diagnosis of DR was reported in Supplementary Appendix SA.

To eliminate the impact due to major known confounding bias caused by the demographic and clinical characteristics of participants and improve the stability of our findings, a PSM approach was applied in the design process (Rosenbaum and Rubin, 1983; Li et al., 2020). We successfully matched 69 pairs of T2DM patients with DR (case) and without DR (control) for primary analysis based on the propensity score including age, gender, BMI, and HbA1c, and the nearest neighbor algorithm was used in the matching process at a ratio of 1:1. Health controls were further matched with T2DM patients as the blank control based on age, gender, and BMI. Details of the study design showed in Supplementary Figure S1.

### Demographic and Clinical Data Collection

Standardized structure questionnaires, containing information on age, gender, height, weight, duration of diabetes, occupation, life habits as well as histories of hypertension, tobacco and alcohol consumption, treatment and family, were used to collect all participants’ demographic characteristics by a face-to-face interview. BMI was calculated as weight (kg)/(height (m) ^2^).

Features for clinical manifestation and biochemistry, including fasting blood glucose (FPG), HbA1c, total cholesterol (TC), triglyceride (TG), high-density lipoprotein cholesterol (HDL-C), low-density lipoprotein cholesterol (LDL-C) as well as systolic blood pressure (SBP) and diastolic blood pressure (DBP), were determined by two systematically trained investigators. The associated standardized operation procedures of this study were strictly followed in the process.

### Metabolomics Analysis

The venous blood samples of all participants were gathered after fasting for more than 8 h. The samples were then prepared in conical polypropylene centrifuge tubes and centrifuged (2000 rpm, *4°C, *10 min) for separating the serum. After that, 1.5 ml serum sample was stored using sterile tubes at −86°C deep low-temperature refrigerator for further metabolomic assessment. The sample preprocessing and metabolomic analyses based on UPLC-MS/MS were carefully carried out by a professional technician in the central laboratory of Metware Inc., a professional metabolomics institution in China, and details were provided in Supplementary Appendix SB.

### Data Processing

The raw data from UPLC-MS/MS was obtained by Analyst Software V.1.6.3 and processed in a widely targeted manner using MultiQuant Software to convert, peak detects, retention time correct and peak align. Metabolites with a coefficient of variation (CV) larger than 30% in the quality control (QC) samples were discarded. The metabolites with a missing ratio of over 20% in the cases or controls were also removed. Besides, for those with a missing ratio of less than 20%, they would be separately imputed by half of the lowest detected peak areas. Afterward, log transformation and Pareto scaling were individually utilized to improve the normality of associated data and make them more comparable.

### Sample Size Estimation

The sample size estimation was reported in our previous study (Zuo et al., 2021). Sample size was calculated by t-tests using G*Power software version 3.1.9.2 (http://stats.idre.ucla.edu/other/gpower/). Taking effect size as 0.5, type I error as 0.05, the allocation ratio of 1, each group 64 patients is needed to achieve a power of 0.8 (Supplementary Figure S2).

### Statistical Analysis

Normally or approximately normally distributed continuous variables were described as mean ± SD and compared by the paired *t*-test. Otherwise, variables with obviously skewed distribution would be presented as median (1st quartile, 3rd quartile) and compared by Wilcoxon signed-rank test. Categorical variables were reported as frequency (percentage) and McNemar-Bowker test or Wilcoxon signed-rank test would be applied for the comparisons.

Paired *t*-test with Benjamini-Hochberg false positive rate (FDR) correction was performed to identify the differentially expressed metabolites (DEMs) when comparing DR with DM. Furthermore, to markedly increase the reliability of detected DEMs, a partial least squares discriminant analysis (PLS-DA) model with 1000-times permutation test was constructed to obtain the variable importance for the projection (VIP) of metabolites. Finally, the criteria of DEMs screening were determined as FDR-adjusted q-value < 0.05, fold changes (FC) > 1.2 or FC < 0.8, and VIP >1. Relationships of the top 25 DEMs, which had the lowest q-value, were additionally visualized *via* a hierarchical clustering heatmap depending on the Euclidean distance metric and Ward’s clustering method.

All above-mentioned data management and analysis were jointly implemented by RStudio version 1.2.5042 (^©^ 2009–2020 RStudio, Inc.) and MetaboAnalyst version 5.0 (Xia et al., 2009).

### Metabolic Pathway Analysis

The metabolic pathway analysis (MetPA) algorithms included hypergeometric test for over-representation analysis and out-degree centrality for pathway topology analysis (Xia and Wishart, 2010). The metabolite background set was defined as the Kyoto Encyclopedia of Genes and Genomes (KEGG) database (Kanehisa et al., 2021), which was one of the most useful metabolomics databases worldwide. Metabolic pathways with hypergeometric test *p*-value less than 0.05 were considered to be disturbed and were further interpreted using KEGG Mapper (https://www.genome.jp/kegg/mapper /). Furthermore, the DR-altered KEGG global metabolic network, which mapped DEMs into the *ko01100* pathway of KEGG database, was presented using iPath version 3.0 to further determine whether MetPA was affected by the background set (Darzi et al., 2018).

Because above MetPA would ignore the metabolites with weak individual effects but strong synergistic variations, we further applied Chemical Similarity Enrichment Analysis (Barupal and Fiehn, 2017), a novel pathway mapping method based on chemical similarity, to compensate for the shortcomings of the above methods. This method can screen the key metabolites and takes the PubChem compound database as background set, which is the largest available compound repository for free (Barupal et al., 2012). The restricted cubic spline (RCS) regression model was applied to assess the associations between key metabolites and DR.

### Integrated Pathway Analysis

Since no single biochemical platform could cover whole metabolites that existed in humans, integrated pathway analysis need to be implemented for roundly exploration of DR-disordered metabolism in multiplatform. MetaMapp was utilized to generate a comprehensively DR-disturbed metabolic network by integrating biochemical pathway and chemical relationships from KEGG and PubChem database (Barupal et al., 2012), and draw by Cytoscape (Saito et al., 2012; Fan et al., 2020). Cytoscape was broadly utilized in omics network visualization. The principles of ChemRICH and MetaMapp were provided in Supplementary Appendix SC.

## Results

### Characteristics of the Study Participants

A total of 69 pairs of DM and DR, including 60 NPDR (9 mild, 31 moderate, and 20 severe) and 9 PDR, were included in the current study. The demographic and clinical variables of participants were described in Table 1. As compared to DM, DR patients tended to have a longer duration of T2DM (*p* = 0.002) and higher SBP (*p* = 0.003). DR patients were more likely to have vision damage (*p* = 0.005) than DM patients. There was no statistical difference in insulin treatment history between two groups (*p* = 0.093).

**TABLE 1:** Demographics and clinical indicators of participants included in the study.

Variable	DM	DR	P
Continuous variable			
Age, years	53.0(48.0,61.0)	56.0(51.0,65.0)	0.022
BMI, Kg/m2	24.4 ± 3.2	24.6 ± 3.5	0.773
FPG, mmol/L	8.4(6.9,12.0)	8.5(6.3,10.2)	0.225
HbA1c, %	10.1 ± 2.3	9.9 ± 1.9	0.500
LDL, mmol/L	2.7 ± 1.0	2.6 ± 1.1	0.617
HDL, mmol/L	1.0(0.8,1.3)	1.1(0.9,1.3)	0.703
TG, mmol/L	1.6(1.0,2.2)	1.4(1.0,1.9)	0.184
TC, mmol/L	4.7 ± 1.1	4.5 ± 1.4	0.337
SBP, mmHg	124(118,139)	135(122,148)	0.003
DBP, mmHg	79(74,86)	76(70,85)	0.449
Duration of diabetes, years	8.0(4.0,13.0)	12.0(8.0,17.0)	0.002
Category variable, n/N			
Gender	—	—	0.625
Male	38/69	36/69	—
Female	31/69	33/69	—
Occupation	—	—	0.825
Manual workers	31/65	34/64	—
Mental worker	15/65	11/64	—
Both	19/65	19/64	—
Center	—	—	0.074
Wenzhou	36/69	48/69	—
Hefei	33/69	21/69	—
Hypertension	—	—	0.078
No	47/66	37/65	—
Yes	19/66	28/65	—
Smoking habits	—	—	0.530
Non-smokers	41/66	36/65	—
Ex-smokers	6/66	8/65	—
Current smokers	19/66	21/65	—
Alcohol consumption	—	—	0.921
Non-drinkers	33/66	29/65	—
Ex-drinkers	3/66	9/65	—
Current drinkers	30/66	27/65	—
Three-generation family history	—	—	0.556
No	33/63	29/66	—
Yes	30/63	37/66	—
Ever insulin therapy	—	—	0.093
No	46/65	55/65	—
Yes	19/65	10/65	—
Heel pain	—	—	0.134
No	55/66	46/65	—
Yes	11/66	19/65	—
Vision loss	—	—	0.005
No	37/66	19/65	—
Yes	29/66	46/65	—

Abbreviations: DM, type 2 diabetes mellitus (T2DM) without diabetic retinopathy; DR, T2DM with diabetic retinopathy; BMI, body mass index; FPG, fasting plasma glucose; HbA1c, glycated hemoglobin; LDL, low density lipoprotein; HDL, high density lipoprotein; TG, triglyceride; TC, total cholesterol; SBP, systolic blood pressure; DBP, diastolic blood pressure.

### Impact of DR on Serum Metabolic Profile

**FIGURE 1:**
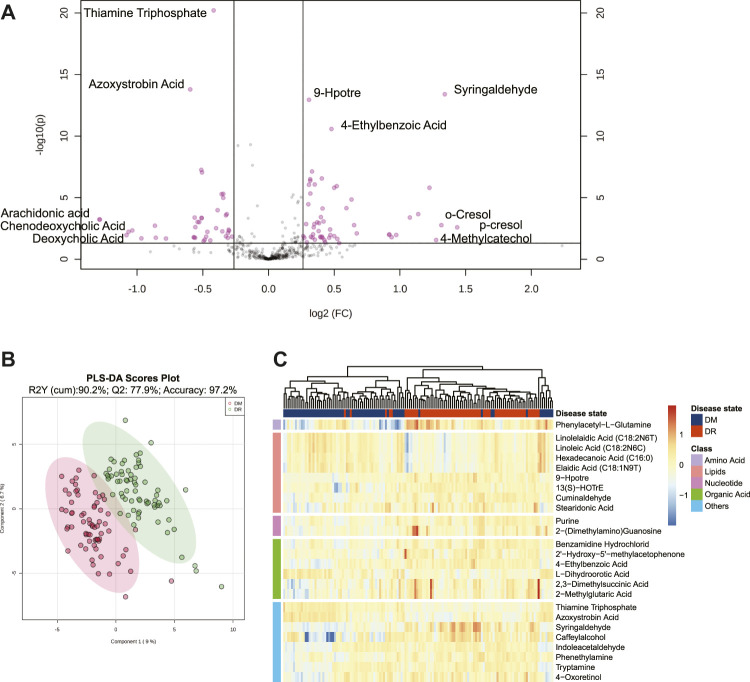
**(A)** Volcano plot of metabolites between DM and DR patients (FDR <0.05; fold changes >1.2 or <0.8). **(B)** PLS-DA score plot. The model was established using three principal components. Cumulative R2 archived 90.2%, Q2 achieved 77.9%, and accuracy achieved 97.2% with permutation test *p*-values less than 0.001. **(C)** Heatmap for intensities of top 25 differentially expressed metabolites between DM and DR patients with the smallest paired *t*-test FDR *q* value. Euclidean distance metric and Ward’s clustering method were applied for the hierarchical clustering. Red represents increased intensities and blue decreased intensities. Abbreviations: DM, type 2 diabetes mellitus (T2DM) without diabetic retinopathy; DR, T2DM with diabetic retinopathy; PLS-DA, partial least squares discriminant analysis; FDR, false discovery rate.

A total of 613 identified metabolites, including 318 in positive ion modes and 295 in negative ion modes, were detected *via* the UPLC-MS/MS system. After the necessary data preprocessing, 461 metabolites participated in the subsequent analysis. The principal component analysis demonstrated that QC samples had good consistency (black nodes), which meant the detection system was robust (Supplementary Figure S3). Based on FDR-adjusted q-value < 0.05, and FC > 1.2 or FC < 0.8, 91 metabolites were differentially presented between the DM and DR patients (Figure 1A). The PLS-DA model classified DM and DR patients clearly, which was concluded that DR induced a significant metabolism disorder (Figure 1B). Furthermore, based on VIP >1, 89 metabolites were determined as DEMs. Among them, 34 were under-regulated and the other 55 were increased in DR when comparing with DM (Supplementary Table S1). Based on the top 25 DEMs with the highest statistical difference, the Hierarchical clustering heatmap also exhibited clearly distinct patterns between DR and DM, the most of which was lipids (Figure 1C).

### Metabolic Pathway Analysis

**FIGURE 2:**
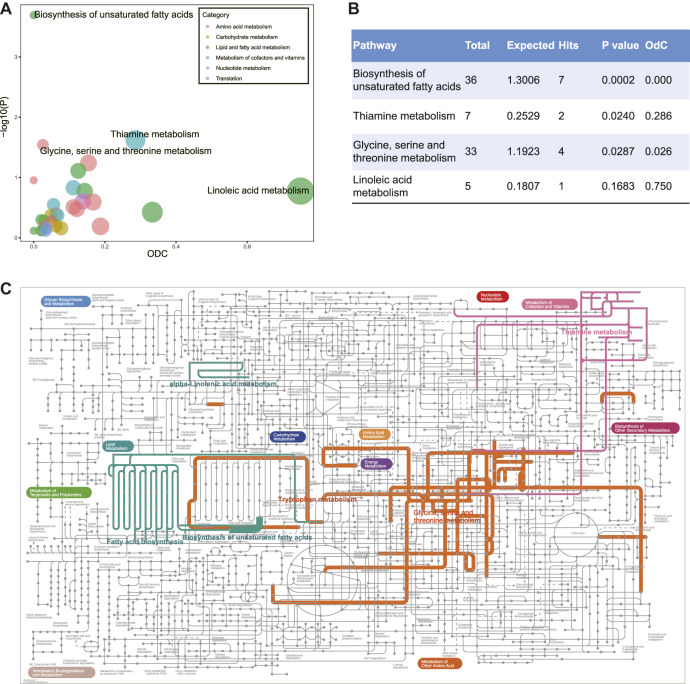
**(A)** Metabolic pathway analysis of differentially expressed metabolites between DM and DR patients. *Y*-axis shows -lg(p) calculated by hypergeometric test using over-representation analysis. *X*-axis and the size show out-degree centrality using pathway topology. The color represents different categories of metabolic pathways. **(B)** Statistic table for enriched metabolic pathways. **(C)** KEGG global metabolic network highlighting DR altered pathways (*p*-value < 0.05). The thickness of the line represents the number of enriched metabolites in the pathway. Abbreviations: DM, type 2 diabetes mellitus (T2DM) without diabetic retinopathy; DR, T2DM with diabetic retinopathy; KEGG, Kyoto Encyclopedia of Genes and Genomes database.

To better understand the biological meaning of DR-related metabolic pathways, we performed the metabolic pathway analysis of 89 DEMs (Figure 2A, Supplementary Table S2). The biosynthesis of unsaturated fatty acids, thiamine metabolism, and glycine, serine and threonine metabolism were detected as the significantly enriched pathways, and the hub metabolite (linoleate) of linoleic acid metabolism was manifestly decreased which caused out-degree centrality equals to 0.75 (Figure 2B). The particular biological annotations of the above four pathways were provided in Supplementary Figures S4–S7. These interpretations reported that the biosynthesis of unsaturated fatty acids in DR was evidently decreased in metabolites of *ω*-6 and *ω*-3 PUFAs families; the elevation of l-cysteine and reduction in thiamine triphosphate constitutes an overt alteration of thiamine metabolism; the glycine, serine and threonine metabolism was also remarkably altered by increased l-cysteine and decreased *ß*-hydroxypyruvic acid, creatine, and sarcosine. In addition, the annotations of KEGG global metabolic network further showed that tryptophan metabolism, fatty acid biosynthesis, and alpha-linolenic acid metabolism were enriched in DR patients (Figure 2C, Supplementary Table S3).

**FIGURE 3:**
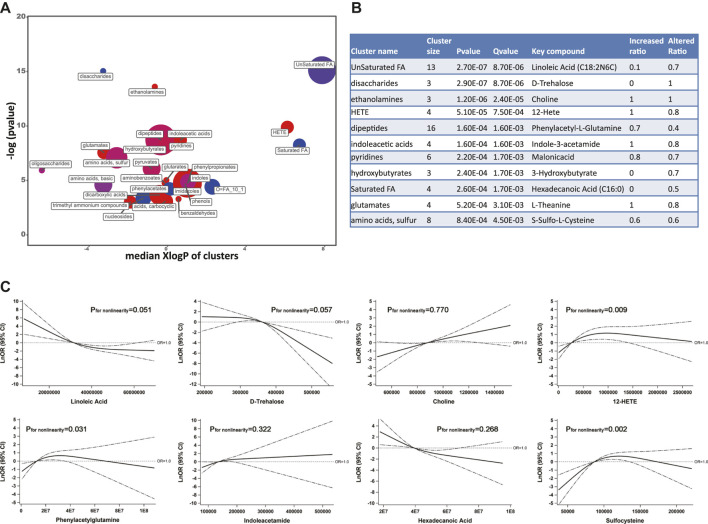
**(A)** ChemRICH analysis showed the most significantly altered metabolite clusters based on chemical similarity. Cluster size indicates the number of metabolites in each cluster. The proportion of increased or decreased metabolites compared to DM patients are shown by color (red = increased, purple = partly decreased, blue = decreased). Chemical enrichment statistics were calculated by the Kolmogorov–Smirnov test and only enrichment clusters with *p* < 0.05 are shown in the bubble plot. **(B)** Statistics table for metabolite clusters (adjusted q value <0.01). **(C)** Associations of key metabolites with the odds (natural log-transformed) of DR after adjusting for systolic blood pressure, duration of diabetes, and insulin treatment history. Abbreviations: ChemRICH, Chemical Similarity Enrichment Analysis for Metabolites; DM, type 2 diabetes mellitus (T2DM) without diabetic retinopathy; DR, T2DM with diabetic retinopathy; KEGG, Kyoto Encyclopedia of Genes and Genomes database.

Based on the threshold-free chemical similarity metric, ChemRICH enriched all PubChem-identified metabolites and DR-altered metabolic clusters were summarized in Figure 3A. Among all the altered metabolic clusters, 11 clusters were considered as significantly perturbed, taking FDR-adjusted q value < 0.01 (Figure 3B). After adjusting for SBP, duration of diabetes, and insulin treatment history, the RCS model showed that the key metabolites of the eight metabolic clusters remained statistically associated with DR, including unsaturated fatty acids (linoleic acid as key compound, negative linear trend of DR with linoleic acid), disaccharides (trehalose, negative linear trend), ethanolamines (choline, positive linear trend), HETE (hydroxyeicosatetraenoic acid, 12-HETE, non-linear trend), dipeptides (phenylacetylglutamine, non-linear trend), indoleacetic acids (indoleacetamide, positive linear trend), saturated fatty acids (hexadecanoic acid, negative linear trend), and amino acids, sulfur (sulfocysteine, negative non-linear trend) (Figure 3C).

### Integrated Pathway Analysis

**FIGURE 4:**
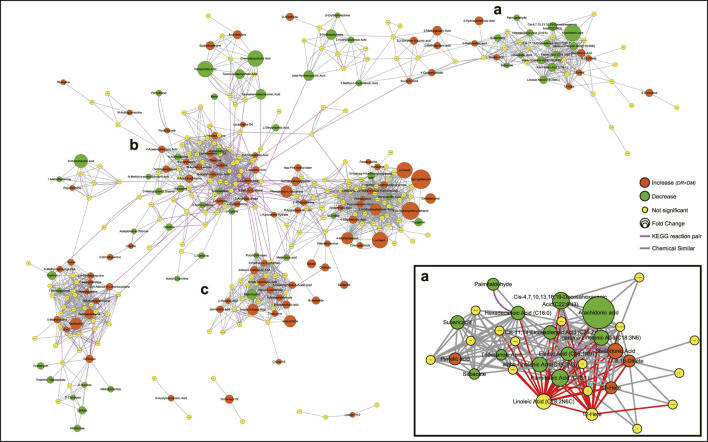
Metabolic network visualizing by MetaMapp. Orange nodes indicate increased metabolites in DR patients compared to DM patients, while the green nodes indicate a decrease. Node size indicates the magnitude of fold change. Purple edges denote KEGG reactant pair links, and grey edges symbolize Tanimoto chemical similarity over 700. Module a mainly includes PUFAs and their derivatives, module b mainly includes amino acids, module c includes indole and its derivatives. Abbreviations: DR, type 2 diabetes mellitus (T2DM) with diabetic retinopathy; DM, T2DM without diabetic retinopathy; KEGG, Kyoto Encyclopedia of Genes and Genomes database.

Based on integrated pathway analysis, all identified metabolites were mapped to the KEGG and PubChem database and the DR-altered metabolic network was generated by MetaMapp (Figure 4). The integrated network revealed that DR-altered metabolites were mainly gathered into several modules by chemical similarity, including PUFAs and their derivatives (a), amino acids (b), and indole and its derivatives (c). According to module a, most of *ω*-6 PUFAs, like linoleic acid (LA), *γ*-linoleic acid (GLA), arachidonic acid (AA), et al., and *ω*-3 PUFAs, like *a*-linoleic acid (ALA), docosahexaenoic acid (DHA), eicosapentanoic acid (EPA), et al., expressed a decreasing trend in DR patients while the metabolites of *ω*-6 PUFAs (12-/15-HETE) were significantly up-regulated. Module b was the central hub in the whole integrated network and was linked to other modules through KEGG reactant pairs. Many amino acids in module b related to glutamate pathways expressed abnormally, such as l-glutamine, l-glutamic acid, l-theanine, et al. Furthermore, the elements of cysteine-related pathways, like l-cystathionine, S-adenosyl-l-homocysteine, l-cystine, l-cysteine, et al., was also disturbed significantly in the DR-altered network. Module c revealed that most indoles had increasing trends in DR patients. The same trend applies to nucleotides and their derivatives.

### Meta-Data Comparison With Previous Study

To confirm the validity of this serum metabolomics study, the results were compared with previous DR blood metabolomics studies (Table 2). Consistent with previous studies, this study showed that fatty acid metabolism (especially PUFAs and their derivatives), amino acid metabolism (especially glycine, serine and threonine metabolism, and glutamate and cysteine-related pathways), and nucleotide-related pathways were significantly perturbed in DR (Figure 4; Table 2). Meanwhile, serum creatine was decreased in DR compared with DM, which was consistent with a PDR vitreous metabolomics study(Tomita et al., 2021). We did not see significant associations between DR and the following metabolites that were demonstrated previously: pyruvic acid, *trans*-oleic acid, hydroxybutyric acid, deoxyribonic acid, erythritol, gluconic acid, ribose, maltose, fumaric acid, uridine, cytidine, 2-piperidone. In contrast, most of PUFAs were modulated in the opposite direction in our study compared with Xuan’s study, while the derivatives (12-/15-HETE and 8,15-DiHETE) were modulated in the same direction (Xuan et al., 2020). We noticed that thiamine metabolism was significantly altered in DR (Figure 2). We also found the following unreported metabolites that were significantly different between DR and DM serum samples: trehalose, choline, indoleacetic acids.

**TABLE 2:** Summary of published studies on blood metabolomics of diabetic retinopathy.

Authors	Year	Platform	Matching	Case	Control	Patients	Biomarker (up)	Biomarker (down)	Pathways implicated
Li et al. (Li et al., 2011)	2011	GC-MS (Plasma)	—	88 type 2 diabetes of different stages of DR	—	Chinese	Not validated pyruvic acid, l-aspartic acid	Not validated arachidonic acid, *trans*-oleic acid, linoleic acid, Β-hydroxybutyric acid	—
Chen et al. (Chen et al., 2016)	2016	GC-MS (Plasma)	HbA1c	40 type 2 diabetes with moderate NPDR	40 type 2 diabetes without DR	Singaporeans of South Indian	2-deoxyribonic acid, 3,4-dihydroxybutyric acid, erythritol, gluconic acid, ribose	Maltose	Pentose phosphate pathway
Rhee et al. (Rhee et al., 2018)	2018	GC-MS, UPLC-MS (Plasma)	Age, sex	72 type 2 diabetes with NPDR and 52 type 2 diabetes with PDR	74 type 2 diabetes without DR	Korean	Glutamine, glutamine/glutamic acid	Glutamic acid	—
Zhu et al. (Zhu et al., 2019)	2019	LC-MS (Plasma)	—	21 type 2 diabetes with PDR	21 type 2 diabetes without DR	Chinese	Not validated fumaric acid, uridine, acetic acid, cytidine	—	Alanine, aspartate and glutamate metabolism, caffeine metabolism, beta-alanine metabolism, purine metabolism, cysteine and methionine metabolism, sulfur metabolism, sphingosine metabolism, arginine and proline metabolism
Xuan et al. (Xuan et al., 2020)	2020	GC-MS, LC-MS (Serum)	Age, sex	350 type 2 diabetes of different stages of DR	111 type 2 diabetes without DR	Chinese	12-HETE, 2-piperidone	—	Energy metabolism, amino acid metabolism, lipid metabolism
Zuo et al. (Zuo et al., 2021)	2021	UPLC-ESI-MS/MS (Serum)	Age, sex, BMI, HbA1c	46 type 2 diabetes of different stages of DR	46 type 2 diabetes without DR	Chinese	Phenylacetylglutamine, nicotinuric acid, ornithine	Linoleic acid	linoleic acid metabolism, alanine, aspartate and glutamate metabolism, phenylalanine metabolism

Abbreviations: GC-MS, gas chromatography-mass spectrometry; UPLC-MS, ultra-performance liquid chromatography-mass spectrometry; LC-MS, liquid chromatography-mass spectrometry; UPLC-ESI-MS/MS, ultra-performance liquid chromatography-electrospray ionization-tandem mass spectrometry; HbA1c: glycated hemoglobin; BMI, body mass index; DR, type 2 diabetes mellitus with diabetic retinopathy; NPDR, non-proliferative diabetic retinopathy; PDR, proliferative diabetic retinopathy.

## Discussion

In this PSM-based case-control study, we comprehensively described DR-disrupted metabolic pathways *via* metabolic pathway analysis based on KEGG and PubChem database. We corroborated the associations of some pathways with DR that were reported previously, including biosynthesis of PUFAs, glycine, serine and threonine metabolism, glutamate and cysteine-related pathways, and nucleotide-related pathways (Li et al., 2011; Rhee et al., 2018; Xuan et al., 2020; Zuo et al., 2021). We also found some novel metabolic clusters associated with DR including thiamine metabolism, tryptophan metabolism, disaccharides (trehalose as key compound), ethanolamines (choline), and indoleacetic acids (indoleacetamide). Contrary to previous studies, we did not observe energy metabolism changes, possibly due to adjustment for HbA1c and BMI. Our work also reveals that it is impossible to study the metabolic alterations of diseases comprehensively based on a single biological knowledge database at present, so integrated pathway analysis may be an ideal remedy.

Thiamine metabolism is enriched in this study, mainly due to the significantly reduced thiamine triphosphate (ThTP) and elevated l-cysteine in DR. ThTP exists as the non-coenzyme form of vitamin B1 in all living organisms (Bettendorff and Wins, 2009). Although the mechanism is currently unclear, ThTP is considered as the allosteric activator of glutamate dehydrogenase (GDH), which promotes the metabolism of glutamate to alpha ketoglutarate (essential substances for the tricarboxylic acid cycle) (Bunik et al., 2016). The growth of serum glutamate proves this opinion. In the other case, ThTP has a specific neurophysiological role and can phosphorylate rapsyn, which may be linked to the facilitation of acetylcholinergic neurotransmission (Bettendorff and Wins, 2009). It partly explains the increased choline in DR. Previous studies have reported the association between cysteine and DR (Zhu et al., 2019; Zuo et al., 2021).

Tryptophan metabolism is one of the most disturbed metabolic pathways according to KEGG global metabolic network. In tryptophan metabolism, indole and its derivatives are increased in DR patients (Figure 4) and indoleacetic acids are significantly disturbed (Figure 3). The fraction of tryptophan reaching the human colon can be catabolized by the gut bacteria to produce a variety of indole derivatives and release them into the systemic circulation (Platten et al., 2019). Platania *et al.* hypothesized that indole derivatives are innovative molecules endowed with both anti-inflammatory and anti-angiogenic properties which are beneficial in DR treatment and the results supported the hypothesis (Platania et al., 2020). Our results further support the above view and suggest that indole derivatives may inhibit the progression of DR in humans by increasing compensatory activity. A human trial has demonstrated that indoleacetic acid by mouth can reduce blood glucose in diabetic patients (Diengott and Mirsky, 1956). Therefore, indole derivatives have the potential to treat DR.

Trehalose is found to be negatively correlated with DR. Chen *et al.* used to explore the relationship between trehalose and DR but failed because the concentration of trehalose could not be detected in most plasma samples by gas chromatography-mass spectrometry system (Chen et al., 2016). Due to the high sensitivity of the UPLC-MS/MS system, this association could be identified. Trehalose cannot be synthesized in the human body and is mainly used in Asian countries as a food stabilizer (Sokolowska et al., 2021). A randomized control study demonstrates that moderate consumption of trehalose is helpful to maintain glucose homeostasis in healthy people (Yoshizane et al., 2020). Existing evidence reveals that trehalose can effectively govern hyperglycemia of diabetic patients via relieving impaired glucose tolerance, mitigating insulin resistance, and reducing postmeal insulin bursts (Sokolowska et al., 2021). Meanwhile, Taya *et al* (Taya et al., 2009) disclose that trehalose is capable of reducing the production of inflammatory cytokines by protecting IkappaB-alpha reduction *in vivo*. Since hyperglycemia and inflammation are essential pathogenic factors contributing to DR and the safety of trehalose has been confirmed (Sokolowska et al., 2021), it is reasonable that trehalose supplement is beneficial to the prevention and control of DR.

PUFAs and their derivatives-related pathways were significantly altered in serum samples of DR patients. Both *ω*-3 and *ω*-6 series of PUFAs are significantly decreased in DR, which is contrary to a recent study (Xuan et al., 2020). Supplementary Figure S8 showed that this declining trend is stable in each stage of DR. By comparing differences in study design, this opposite result may be caused by the adjustment for HbA1c and BMI. Growing evidence reveals that PUFAs have critical abilities in angiogenesis, regulation of inflammation, and homeostasis maintenance (Elmasry et al., 2019; Xuan et al., 2020; Zuo et al., 2021). They generally work in a two-step reaction sequence. First, PUFAs will be over-released by the action of phospholipase A2 enzyme from the cell membrane lipid layers in a hyperglycemic or hypoxic environment. Second, the released PUFAs are metabolized by either Cytochrome P450, lipoxygenases, or Cyclooxygenases enzymes (Elmasry et al., 2019). After then, the metabolites of *?*-6 PUFAs (e.g., HETE) induce proangiogenic and proinflammatory effects on the promotion of DR, while most of *ω*-6 PUFAs have forceful inhibitory impacts on the progress of DR (Houtsmuller et al., 1981). This is further confirmed epidemiologically by the elevated 12-/15-HETE and reduced PUFAs in DR. On the other hand, the harmful effects of HETEs are offset by the metabolites derived from *ω*-3 PUFAs (Supplementary Figure S9).

### Strengths and Limitations

Our work has several strengths. First, two knowledge databases, including KEGG and PubChem, are integrated to assess the metabolic pathway dysregulation in DR since no single platform can cover all human metabolites so far. Second, the UPLC-MS/MS platform rather than traditional detection strategies is used to efficiently acquire metabolic profiling data, which improves the credibility of our results. Third, the impacts of potential confounding bias are adjusted by the PSM approach, which can achieve an effect similar to multivariable analysis. This study also has some limitations. First, the sample size of our study is not large due to the high cost of metabolomics. Nevertheless, the current sample size is sufficient since it has been thoroughly assessed during study design with the power larger than 0.8. Second, the enzymatic reactions among metabolites cannot be directly calculated to map to the integrated metabolic network. Third, though we reckon that our findings are robust because of careful design, accurate measurements, and comprehensive data analysis, they need to be verified by more prospective cohorts or experimental studies.

In conclusion, the aforementioned results suggest that the metabolism disorder in DR can be better understood through integrating multiple bioinformatics databases. Apart from the known PUFAs metabolism, amino acid metabolism, and nucleotide metabolism, the occurrence and progression of DR is also associated with the disturbance of thiamine metabolism and tryptophan metabolism, decreased trehalose, and increased choline and indole derivatives.

## Data Availability

The datasets presented in this study can be found in online repositories. The names of the repository/repositories and accession number(s) can be found below: MetaboLights database with accession MTBLS4250.
