# Supplementary material for: High-Coverage Serum Metabolomics Reveals Metabolic Pathway Dysregulation in Diabetic Retinopathy: A Propensity Score-Matched Study
Source: Front Mol Biosci. 2022 Mar 17;9:822647. doi: 10.3389/fmolb.2022.822647 (PMC8970305; doi:10.3389/fmolb.2022.822647)
Supplement: Supplementary file 1 [file DataSheet1.docx]

**Supplemental material**

**Appendix A**

**Grade of diabetic retinopathy**

Professional photographers used a digital non-mydriatic fundus camera to obtain bilateral retinal 45-degree images of the macula (centered on the fovea). Fundus photographs were graded preliminarily for the level of diabetic retinopathy (DR) and other fundus lesions by two graders depending on the retinal photographs assessment. If one eye was unavailable for the classification, the other one was graded. DR grading and its severity was determined depending on the worse eye, and each eye was assessed as follows: no apparent DR and any DR, which would be further classified into mild non-proliferative DR (NPDR), moderate NPDR, severe NPDR, and proliferative DR (PDR) according to International Clinical Diabetic Retinopathy Disease Severity Scale. To further investigate the consistency of the diagnosis on DR independently finished by two professional ophthalmologists, an investigation on the interrater reliability was applied using kappa coefficient, a commonly used reliability statistic to estimate interrater reliability in practice. The associated kappa coefficient for two graders’ agreement on fundus photographs were 0.86, which strongly indicated that the diagnosis and grading of DR had excellent agreement in the two ophthalmologists (In statistics, if the kappa coefficient is greater than 0.81, it will be defined as perfect). Few inconsistent samples were graded again by another experienced ophthalmologist of our fundus reading center.

**Appendix B**

**Widely targeted UPLC-MS/MS-based metabolic profiling**

Serum samples were thawed at 4 °C; then, 100 μL of each serum sample was extracted with 300 μL of methanol. The mixture was vortexed and centrifuged (12000 rpm, *4 °C, *10 min); hereafter, the supernatant was extracted into new centrifuge tubes and centrifuged (12000 rpm, *4 °C, *3 min) again. The extracted supernatant was transferred into the UPLC (Shim-pack UFLC SHIMADZU CBM30A) – MS/MS (Applied Biosystems 6500 QTRAP) system for analysis. Quality control (QC) samples were obtained by mixing an equal volume of serum samples and pretreated as described above.

A Waters ACQUITY UPLC system coupled with electrospray ionization (ESI)-mass spectrometer was used for separation and metabolic analysis. The injection volume was 2 μL, the column temperature was set to 40 °C with the flow rate of 0.35 mL/min. The chromatography columns in the positive and negative ion modes were HSS C18 and HSS T3, respectively. Ultra-pure water and acetonitrile (both containing 0.04% formic acid) were utilized as mobile phases A and B. The starting composition was 5% B, which was increased to 95% at 11.0 min for a 1.0 min wash, followed by returning to 5% B in 0.1 min and held until 14 min for a re-equilibration step. For metabolite analysis, the ESI temperature was 500 °C. The mass spectrometry voltage was 5.5 kV. The curtain gas was 25 psi. The parameter of collision-activated dissociation was high. In the triple quadrupole, each ion pair was scanned based on the optimized declustering potential and collision energy. The quality control (QC) samples were inserted into the analysis sequence every 20 samples and analyzed to evaluate the analytical quality. The principal component analysis (PCA) model with DM, DR, and QC samples was utilized to assess the robustness of the detection system (Figure S3A).

**Appendix C**

**Chemical Similarity Enrichment Analysis (ChemRICH)**

ChemRICH is a novel pathway mapping method based on chemical similarity. In ChemRICH, chemical ontologies and structural similarities were applied to cluster metabolites instead of determination by pre-defined metabolic pathway database. Self-contained Kolmogorov-Smirnov (KS) test was performed to calculate the enrichment p-values of identified metabolite clusters, which were generally applied to determine whether a cluster was represented more than expected by chance.

**MetaMapp**

MetaMapp was applied for integrating information from metabolite pathways (KEGG reactant pair database), chemical structures and mass spectral similarity. The metabolites’ encoded chemical structures of MetaMapp were retrieved from the PubChem compound database according to compound identifiers and the NCBI Batch Entrez Utility. The similarity among encoded structures in the MetaMapp was assessed by Tanimoto chemical similarity coefficient (range 0.0 to 1.0), which was represented by the size and 0.7 was defined as the threshold of the similarity among metabolites.


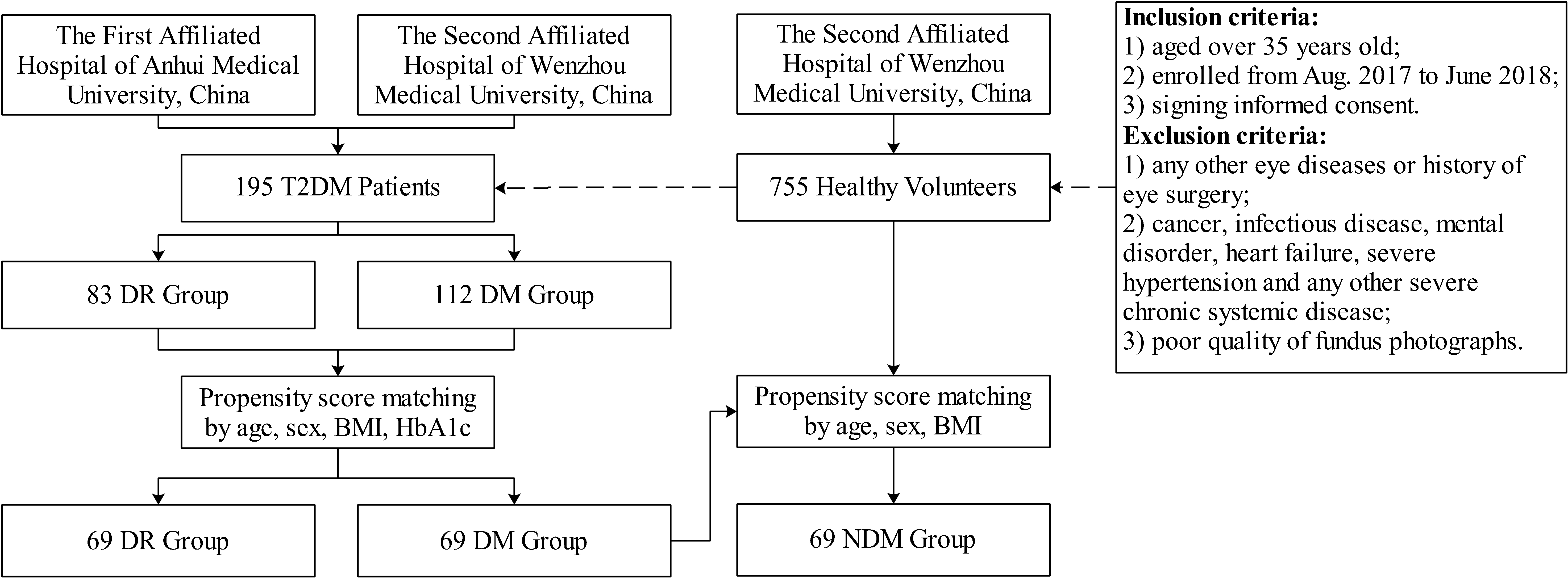


**Figure S1.** The flowchart of the study design.

*Abbreviations: T2DM: type 2 diabetes mellitus; DM: T2DM without diabetic retinopathy; DR: T2DM with diabetic retinopathy; NDM: volunteers without T2DM; BMI: body mass index; HbA1c: glycated hemoglobin.*

| 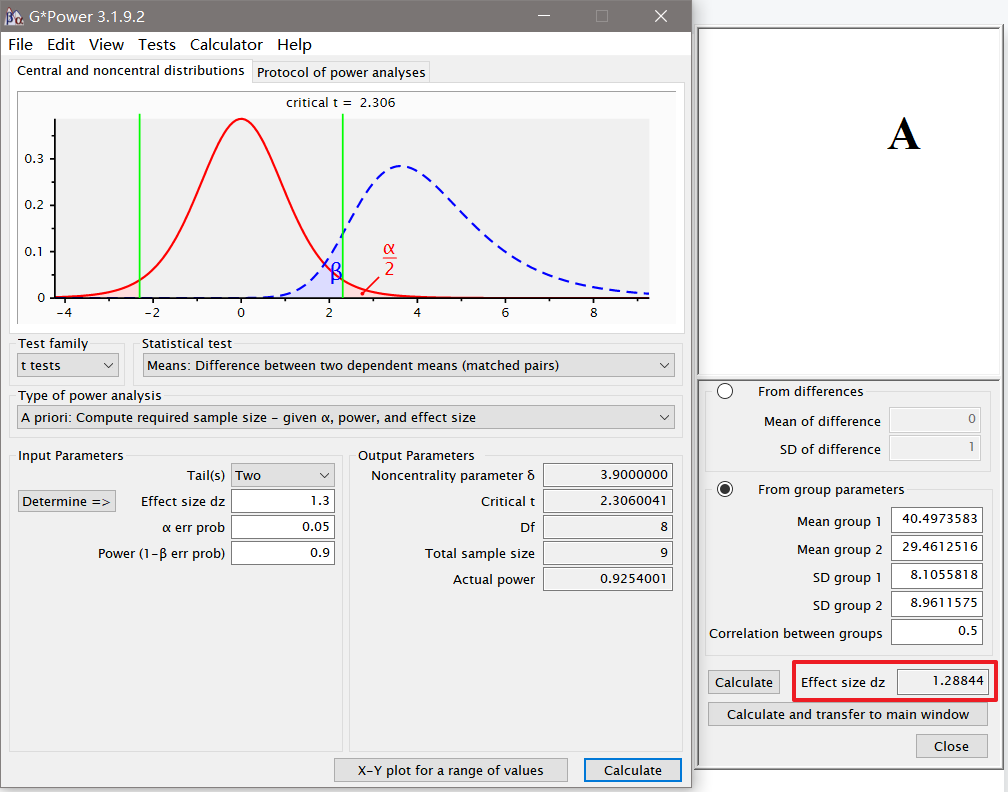 |
| --- |
| 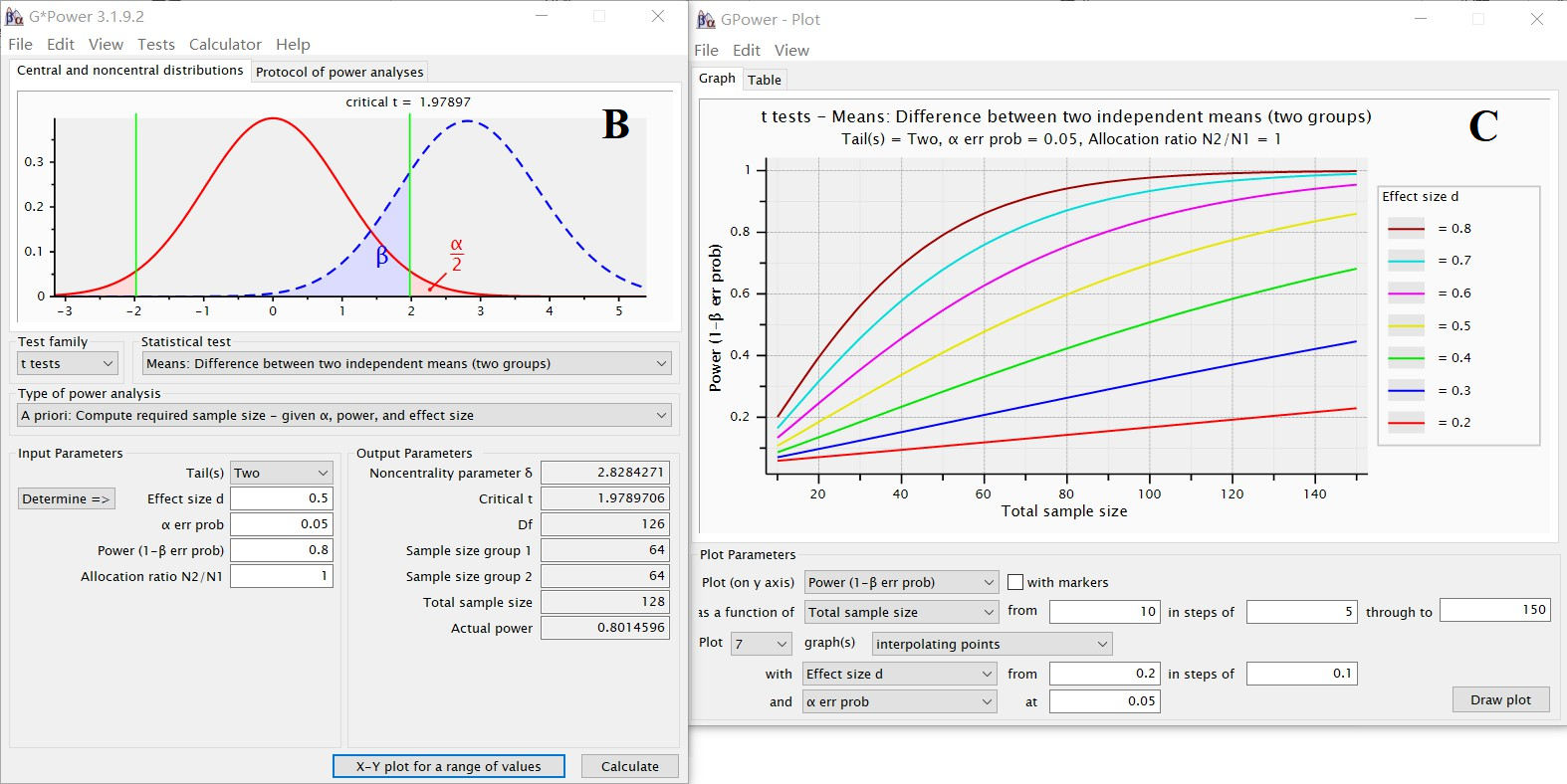 |

**Figure S2.** Power Analysis.

*Note: A: calculation of effect size for post hoc power analysis. B and C: prospective power analysis.*


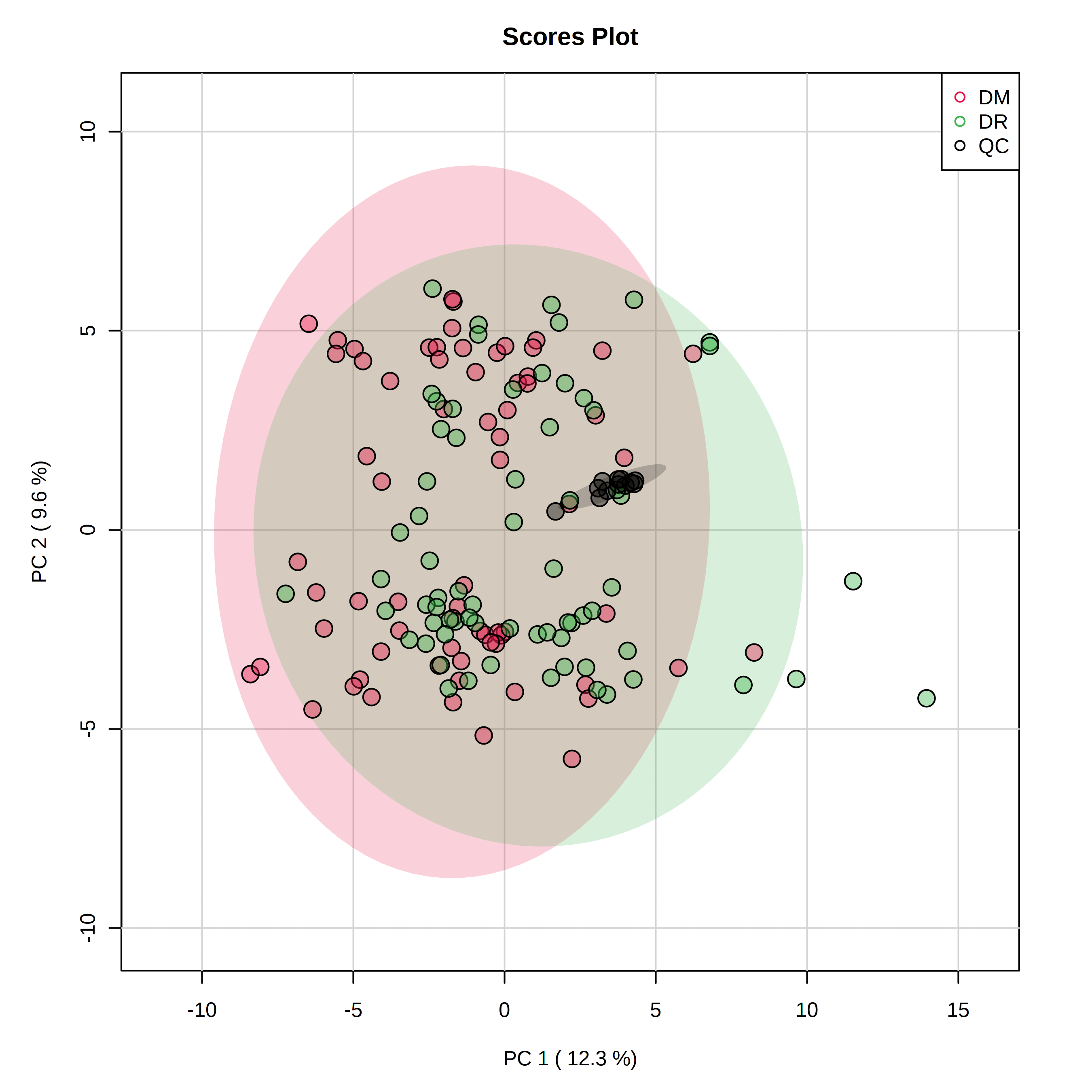


**Figure S3.** PCA score plot of DM, DR, and QC groups. It shows the UPLC-MS/MS platform using in the study is relatively robust.

*Abbreviations: PCA: Principal component analysis; DM: diabetes mellitus; DR: diabetic retinopathy; QC: quality control; UPLC-MS/MS: ultra-performance liquid chromatography-tandem mass spectrometry.*

**Table S1.** Differentially expressed metabolites in diabetic retinopathy patients.

| Metabolites | ION MODE | P value | FDR-adjusted q value | Fold change | VIP |
| --- | --- | --- | --- | --- | --- |
| Thiamine Triphosphate | Negative | 1.32E-23 | 6.08E-21 | 0.75 | 2.4 |
| Azoxystrobin Acid | Positive | 6.93E-17 | 1.60E-14 | 0.66 | 2.53 |
| Syringaldehyde | Positive | 2.57E-16 | 3.96E-14 | 2.83 | 4.03 |
| 9-Hpotre | Negative | 9.72E-16 | 1.12E-13 | 1.24 | 1.87 |
| 4-Ethylbenzoic Acid | Positive | 2.87E-13 | 2.65E-11 | 1.37 | 2.26 |
| Linolelaidic Acid (C18:2N6T) | Negative | 1.08E-09 | 5.52E-08 | 0.71 | 2.13 |
| Phenethylamine | Positive | 1.63E-09 | 7.51E-08 | 1.25 | 1.68 |
| Linoleic Acid (C18:2N6C) | Negative | 2.12E-09 | 8.86E-08 | 0.71 | 2.1 |
| 13(S)-HOTrE | Negative | 7.17E-09 | 2.76E-07 | 1.27 | 1.84 |
| Cuminaldehyde | Positive | 8.87E-09 | 3.15E-07 | 1.24 | 1.59 |
| Benzamidine Hydrochlorid | Positive | 1.28E-08 | 4.22E-07 | 1.24 | 1.61 |
| 2'-Hydroxy-5'-methylacetophenone | Positive | 2.76E-08 | 8.47E-07 | 1.31 | 1.69 |
| Purine | Positive | 3.11E-08 | 8.95E-07 | 1.23 | 1.56 |
| 4-Oxoretinol | Positive | 4.29E-08 | 1.16E-06 | 1.37 | 1.99 |
| Caffeylalcohol | Positive | 6.38E-08 | 1.57E-06 | 1.7 | 3.07 |
| Stearidonic Acid | Positive | 6.49E-08 | 1.57E-06 | 1.41 | 1.91 |
| Indoleacetaldehyde | Negative | 2.00E-07 | 4.62E-06 | 1.21 | 1.51 |
| L-Dihydroorotic Acid | Negative | 2.22E-07 | 4.88E-06 | 0.77 | 1.7 |
| Hexadecanoic Acid (C16:0) | Negative | 2.46E-07 | 5.15E-06 | 0.78 | 1.66 |
| 2,3-Dimethylsuccinic Acid | Negative | 8.34E-07 | 1.42E-05 | 1.64 | 2.12 |
| Tryptamine | negative | 2.23E-06 | 3.43E-05 | 1.21 | 1.45 |
| 2-Methylglutaric Acid | Negative | 4.98E-06 | 7.17E-05 | 1.59 | 2 |
| 2-(Dimethylamino)Guanosine | Positive | 5.43E-06 | 7.59E-05 | 1.39 | 1.68 |
| Elaidic Acid (C18:1N9T) | Negative | 7.68E-06 | 1.04E-04 | 0.76 | 1.61 |
| Sarcosine | Positive | 1.70E-05 | 2.19E-04 | 0.77 | 1.44 |
| Phenylacetyl-L-Glutamine | Negative | 1.71E-05 | 2.19E-04 | 1.86 | 2.7 |
| N6-Succinyl Adenosine | Negative | 3.15E-05 | 3.82E-04 | 1.41 | 1.55 |
| 2-(4-Hydroxyphenyl)ethanol | Negative | 3.47E-05 | 4.10E-04 | 2.58 | 2.56 |
| Palmitoleic Acid (C16:1) | Negative | 3.61E-05 | 4.16E-04 | 0.65 | 1.86 |
| Homo-Gamma-Linolenic Acid (C20:3) | Negative | 3.89E-05 | 4.37E-04 | 0.66 | 1.77 |
| cis-7-Hexadecenoic Acid | Negative | 4.17E-05 | 4.58E-04 | 0.69 | 1.76 |
| Chenodeoxycholic Acid | Negative | 5.91E-05 | 5.97E-04 | 0.42 | 2.66 |
| Deoxycholic Acid | Negative | 5.96E-05 | 5.97E-04 | 0.42 | 2.65 |
| Asp-Phe methyl ester | Positive | 8.75E-05 | 8.58E-04 | 1.34 | 1.59 |
| gamma-Linolenic Acid(C18:3N6) | Negative | 9.84E-05 | 9.45E-04 | 0.7 | 1.78 |
| alpha-Linolenic Acid(C18:3N3) | Negative | 1.03E-04 | 9.65E-04 | 0.71 | 1.76 |
| 2,2-Dimethyl Succinic acid | Negative | 1.12E-04 | 1.02E-03 | 1.37 | 1.46 |
| Lysops 22:5 | Negative | 1.32E-04 | 1.17E-03 | 1.25 | 1.52 |
| Indole-3-acetamide | Positive | 1.61E-04 | 1.40E-03 | 1.25 | 1.28 |
| S-Sulfo-L-Cysteine | Positive | 1.64E-04 | 1.40E-03 | 1.24 | 1.42 |
| Isobutyryl carnitine | Positive | 2.11E-04 | 1.72E-03 | 1.55 | 1.92 |
| o-Cresol | Negative | 2.12E-04 | 1.72E-03 | 2.36 | 2.67 |
| Tauroursodeoxycholic Acid | Negative | 2.40E-04 | 1.91E-03 | 0.64 | 2.16 |
| 3-Methylglutaric acid | Negative | 3.40E-04 | 2.58E-03 | 1.35 | 1.34 |
| p-cresol | Negative | 3.41E-04 | 2.58E-03 | 2.35 | 2.76 |
| Cis-11,14-Eicosadienoic Acid (C20:2) | Negative | 4.13E-04 | 3.02E-03 | 0.75 | 1.46 |
| 10-Formyl-Thf | Positive | 5.37E-04 | 3.87E-03 | 1.54 | 1.57 |
| Imidazoleacetic acid | Negative | 6.60E-04 | 4.61E-03 | 0.48 | 2.34 |
| Creatine | Negative | 7.11E-04 | 4.90E-03 | 0.79 | 1.14 |
| D-Xylonic Acid Lithium Salt | Negative | 7.62E-04 | 5.17E-03 | 1.35 | 1.4 |
| Fosfomycin | Negative | 8.22E-04 | 5.49E-03 | 0.77 | 1.32 |
| Taurochenodesoxycholic Acid | Negative | 9.06E-04 | 5.97E-03 | 0.6 | 2.26 |
| Cis-4,7,10,13,16,19-Docosahexaenoic Acid(C22:6N3) | Negative | 9.24E-04 | 6.00E-03 | 0.66 | 1.54 |
| Arachidonic Acid(C20:4N6) | Negative | 1.16E-03 | 7.40E-03 | 0.79 | 1.24 |
| Sotalol | Positive | 1.29E-03 | 8.02E-03 | 1.47 | 1.8 |
| 5-Hydroxyindole-3-Acetic Acid | Positive | 1.58E-03 | 9.59E-03 | 1.27 | 1.28 |
| 12-Hete | Negative | 1.64E-03 | 9.83E-03 | 1.22 | 1.98 |
| 15-Hete | Negative | 1.74E-03 | 1.03E-02 | 1.23 | 1.98 |
| Hydroxy-Methoxycinnamate | Positive | 1.86E-03 | 1.08E-02 | 15.35 | 2.13 |
| Trans-Zeatin 9-O-Glucoside | Positive | 1.87E-03 | 1.08E-02 | 1.42 | 1.49 |
| Arachidonic acid | Positive | 1.95E-03 | 1.11E-02 | 0.39 | 2.17 |
| 5-Hydroxy-L-Tryptophan | Positive | 2.40E-03 | 1.35E-02 | 1.22 | 1.09 |
| Xanthosine | Negative | 2.42E-03 | 1.35E-02 | 1.92 | 1.48 |
| Subericacid | Positive | 2.81E-03 | 1.47E-02 | 0.8 | 1.05 |
| Indole-2-Carboxylic Acid | Positive | 2.89E-03 | 1.47E-02 | 1.4 | 1.3 |
| L-Aspartic Acid | Negative | 2.94E-03 | 1.47E-02 | 1.29 | 1.32 |
| Maltotriose | Positive | 2.94E-03 | 1.47E-02 | 0.75 | 1.47 |
| N-Acetylneuraminic Acid | Negative | 3.14E-03 | 1.54E-02 | 1.21 | 1.16 |
| N-Phenylacetylglycine | Negative | 3.39E-03 | 1.64E-02 | 1.46 | 2.03 |
| beta-Hydroxypyruvic Acid | Negative | 3.49E-03 | 1.67E-02 | 0.6 | 1.5 |
| 3-Hydroxybutyrate | Negative | 3.66E-03 | 1.71E-02 | 0.6 | 1.49 |
| Bumetanide | Negative | 3.68E-03 | 1.71E-02 | 1.35 | 1.29 |
| S-(5-Adenosy)-L-Homocysteine | Negative | 3.79E-03 | 1.75E-02 | 1.25 | 1.23 |
| Digitoxigenine | Positive | 4.03E-03 | 1.82E-02 | 0.47 | 1.8 |
| L-Cysteine | Negative | 4.11E-03 | 1.82E-02 | 1.28 | 1.34 |
| Indole Carboxylic Acid | Positive | 4.14E-03 | 1.82E-02 | 1.44 | 1.33 |
| N-Acetyl-L-Leucine | Negative | 4.19E-03 | 1.82E-02 | 1.23 | 1.13 |
| Malonicacid | Negative | 4.23E-03 | 1.82E-02 | 0.61 | 1.47 |
| 3-Hydroxy-4-methoxyphenylacetic acid | Negative | 4.40E-03 | 1.86E-02 | 0.8 | 1.11 |
| 4-Hydroxy-6-methyl-2-pyrone | Negative | 4.83E-03 | 2.02E-02 | 0.49 | 2.08 |
| (3,4-Dimethoxyphenyl) Acetic Acid | Negative | 5.05E-03 | 2.10E-02 | 0.74 | 1.77 |
| N-Acetylmethionine | Negative | 5.46E-03 | 2.25E-02 | 1.41 | 1.39 |
| 5-Methyluridine | Positive | 6.56E-03 | 2.61E-02 | 1.27 | 1.03 |
| Lactose | Positive | 6.65E-03 | 2.62E-02 | 0.73 | 1.41 |
| 4-Methylcatechol | Negative | 7.07E-03 | 2.76E-02 | 1.86 | 2.29 |
| 5-Hydroxyhexanoic Acid | Negative | 7.24E-03 | 2.80E-02 | 1.53 | 1.33 |
| Isoquinoline | Positive | 1.14E-02 | 4.12E-02 | 1.94 | 1.23 |
| 3-Methyladipic acid | Negative | 1.21E-02 | 4.25E-02 | 1.21 | 1.02 |
| Deoxyguanosine | Positive | 1.50E-02 | 4.98E-02 | 1.58 | 1.42 |

**Table S2.** The diabetic retinopathy disturbed metabolic pathways detected by metabolic pathway analysis.

| Pathway | Total | Expected | Hits | P value | Pathway Impact |
| --- | --- | --- | --- | --- | --- |
| Biosynthesis of unsaturated fatty acids | 36 | 1.30 | 7 | <0.001 | 0.00 |
| Thiamine metabolism | 7 | 0.25 | 2 | 0.024 | 0.29 |
| Glycine, serine and threonine metabolism | 33 | 1.19 | 4 | 0.029 | 0.03 |
| Tryptophan metabolism | 41 | 1.48 | 4 | 0.057 | 0.15 |
| alpha-Linolenic acid metabolism | 13 | 0.47 | 2 | 0.078 | 0.13 |
| Histidine metabolism | 16 | 0.58 | 2 | 0.111 | 0.00 |
| Pantothenate and CoA biosynthesis | 19 | 0.69 | 2 | 0.148 | 0.11 |
| Synthesis and degradation of ketone bodies | 5 | 0.18 | 1 | 0.168 | 0.14 |
| Linoleic acid metabolism | 5 | 0.18 | 1 | 0.168 | 0.75 |
| Aminoacyl-tRNA biosynthesis | 48 | 1.73 | 3 | 0.249 | 0.14 |
| Taurine and hypotaurine metabolism | 8 | 0.29 | 1 | 0.256 | 0.17 |
| One carbon pool by folate | 9 | 0.33 | 1 | 0.283 | 0.06 |
| Phenylalanine metabolism | 10 | 0.36 | 1 | 0.309 | 0.11 |
| Cysteine and methionine metabolism | 33 | 1.19 | 2 | 0.336 | 0.12 |
| Arachidonic acid metabolism | 36 | 1.30 | 2 | 0.376 | 0.33 |
| Arginine biosynthesis | 14 | 0.51 | 1 | 0.404 | 0.06 |
| Butanoate metabolism | 15 | 0.54 | 1 | 0.426 | 0.05 |
| Nicotinate and nicotinamide metabolism | 15 | 0.54 | 1 | 0.426 | 0.07 |
| Primary bile acid biosynthesis | 46 | 1.66 | 2 | 0.502 | 0.02 |
| Fatty acid biosynthesis | 47 | 1.70 | 2 | 0.513 | 0.03 |
| beta-Alanine metabolism | 21 | 0.76 | 1 | 0.541 | 0.05 |
| Galactose metabolism | 27 | 0.98 | 1 | 0.633 | 0.06 |
| Glutathione metabolism | 28 | 1.01 | 1 | 0.646 | 0.03 |
| Alanine, aspartate and glutamate metabolism | 28 | 1.01 | 1 | 0.646 | 0.19 |
| Purine metabolism | 65 | 2.35 | 2 | 0.692 | 0.03 |
| Glyoxylate and dicarboxylate metabolism | 32 | 1.16 | 1 | 0.696 | 0.08 |
| Arginine and proline metabolism | 38 | 1.37 | 1 | 0.757 | 0.03 |
| Fatty acid elongation | 39 | 1.41 | 1 | 0.766 | 0.00 |
| Fatty acid degradation | 39 | 1.41 | 1 | 0.766 | 0.02 |
| Pyrimidine metabolism | 39 | 1.41 | 1 | 0.766 | 0.03 |

# *
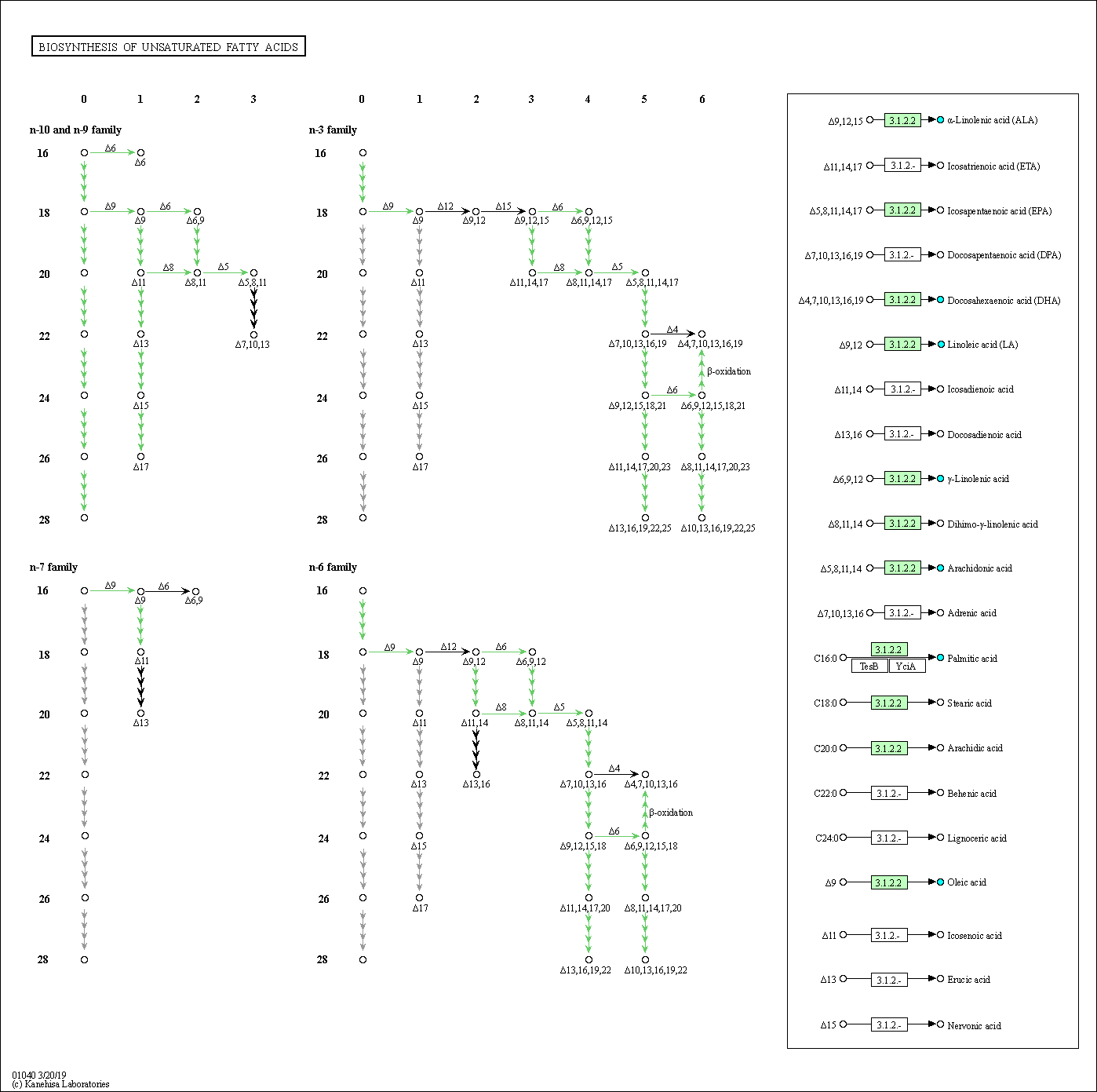
*

**Figure S4.** DR-altered metabolites in biosynthesis of unsaturated fatty acids. Blue nodes indicate decreased metabolites in DR patients as compared to DM patients.


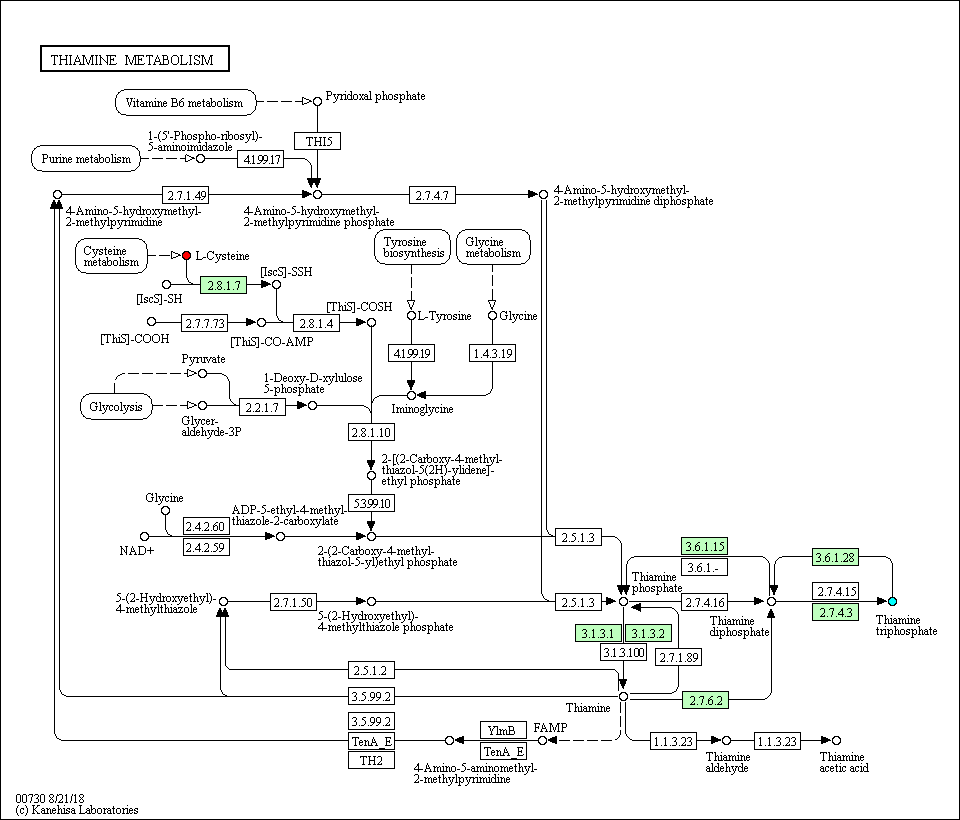


**Figure S5.** DR-altered metabolites in thiamine metabolism. Red nodes indicate increased metabolites in DR patients as compared to DM patients, while the blue indicates a decrease.


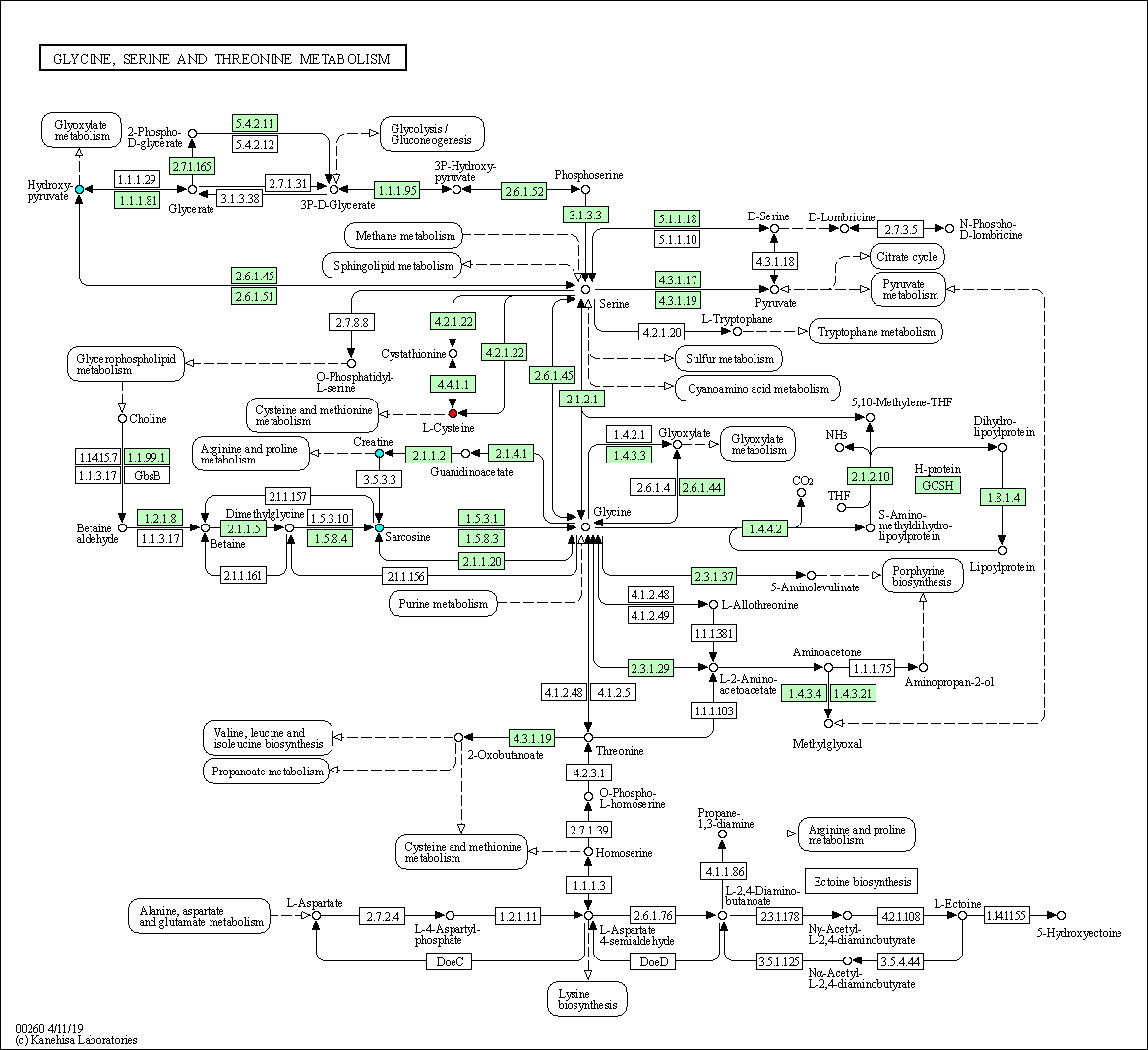


**Figure S6.** DR-altered metabolites in glycine, serine and threonine metabolism. Red nodes indicate increased metabolites in DR patients as compared to DM patients, while the blue indicates a decrease.


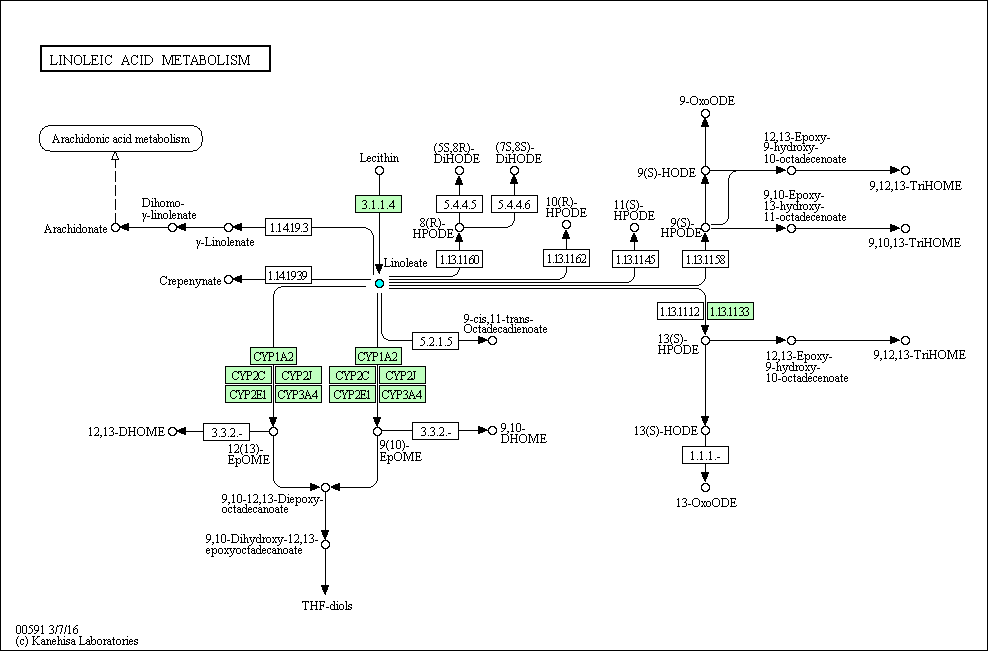


**Figure S7.** DR-altered metabolite in linoleic acid metabolism. The blue indicates a decrease.

**Table S3.** DR altered metabolic pathway (raw p-value < 0.05) of KEGG global metabolic network.

| **Pathway** | **Hits** | **P value** |
| --- | --- | --- |
| Biosynthesis of unsaturated fatty acids | 7 | 0.00000673 |
| Glycine, serine and threonine metabolism | 4 | 0.00447 |
| Thiamine metabolism | 2 | 0.00512 |
| Tryptophan metabolism | 4 | 0.0138 |
| Fatty acid biosynthesis | 2 | 0.0214 |
| alpha-Linolenic acid metabolism | 2 | 0.0305 |

*Abbreviations: KEGG: Kyoto Encyclopedia of Genes and Genomes database; DR: diabetic retinopathy.*

| **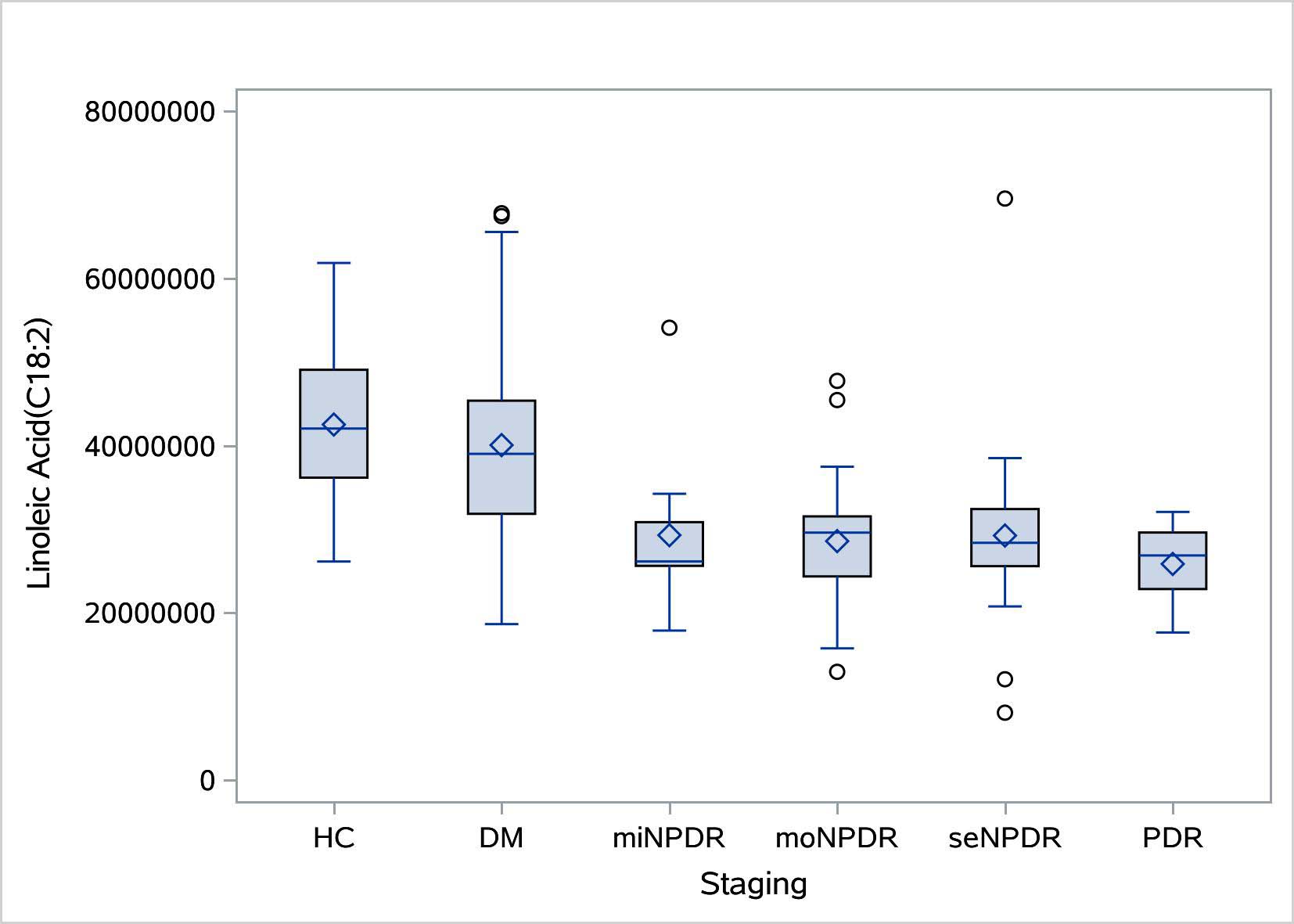** | **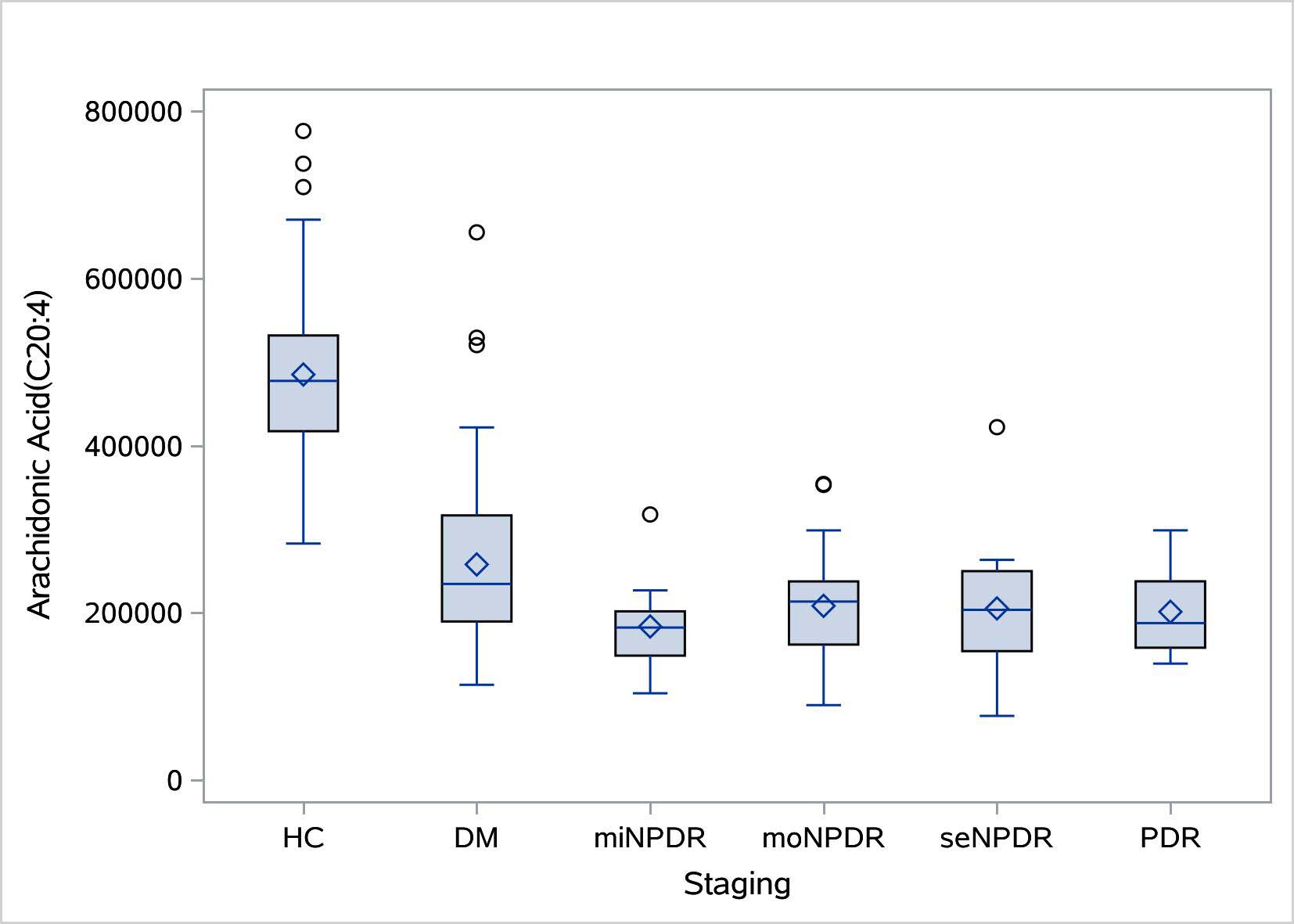** |
| --- | --- |
| **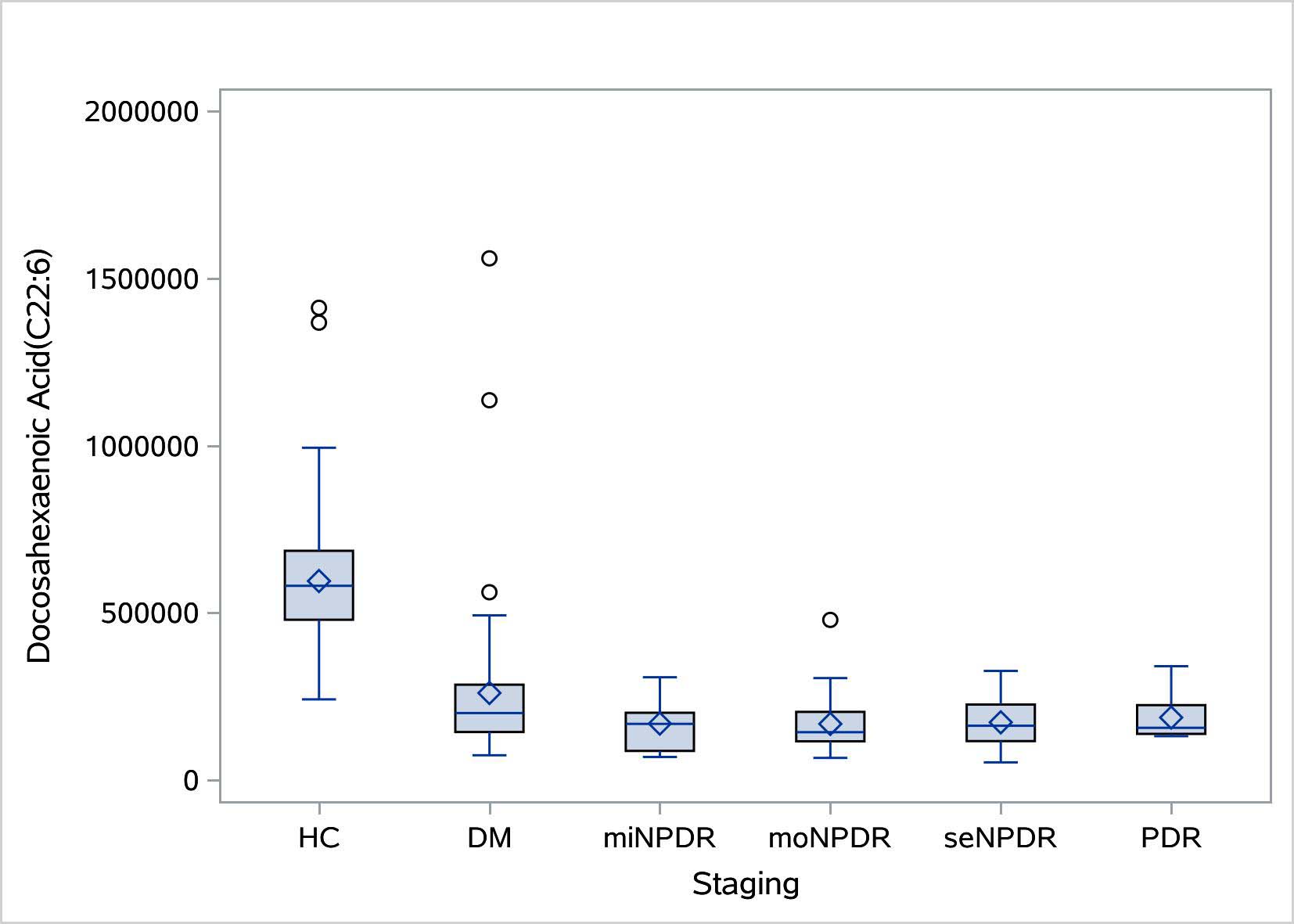** | **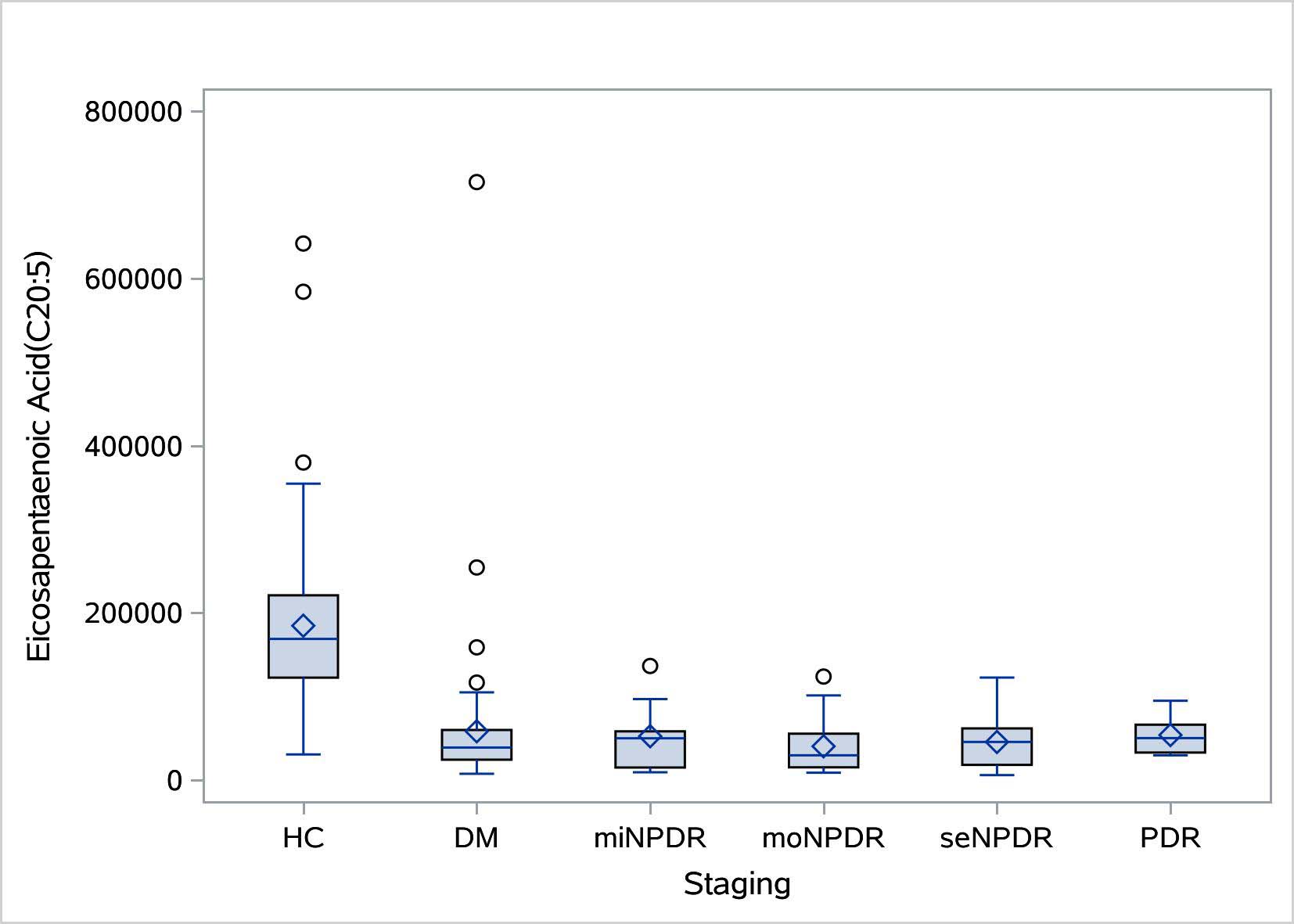** |

**Figure S8.** The boxplots of unsaturated fatty acids in different disease staging.

*Abbreviations: HC: health controls; DM: diabetes mellitus; miNPDR: mild non-proliferative diabetic retinopathy; moNPDR: moderate non-proliferative diabetic retinopathy; seNPDR: severe non-proliferative diabetic retinopathy; PDR:* proliferative *diabetic retinopathy.*


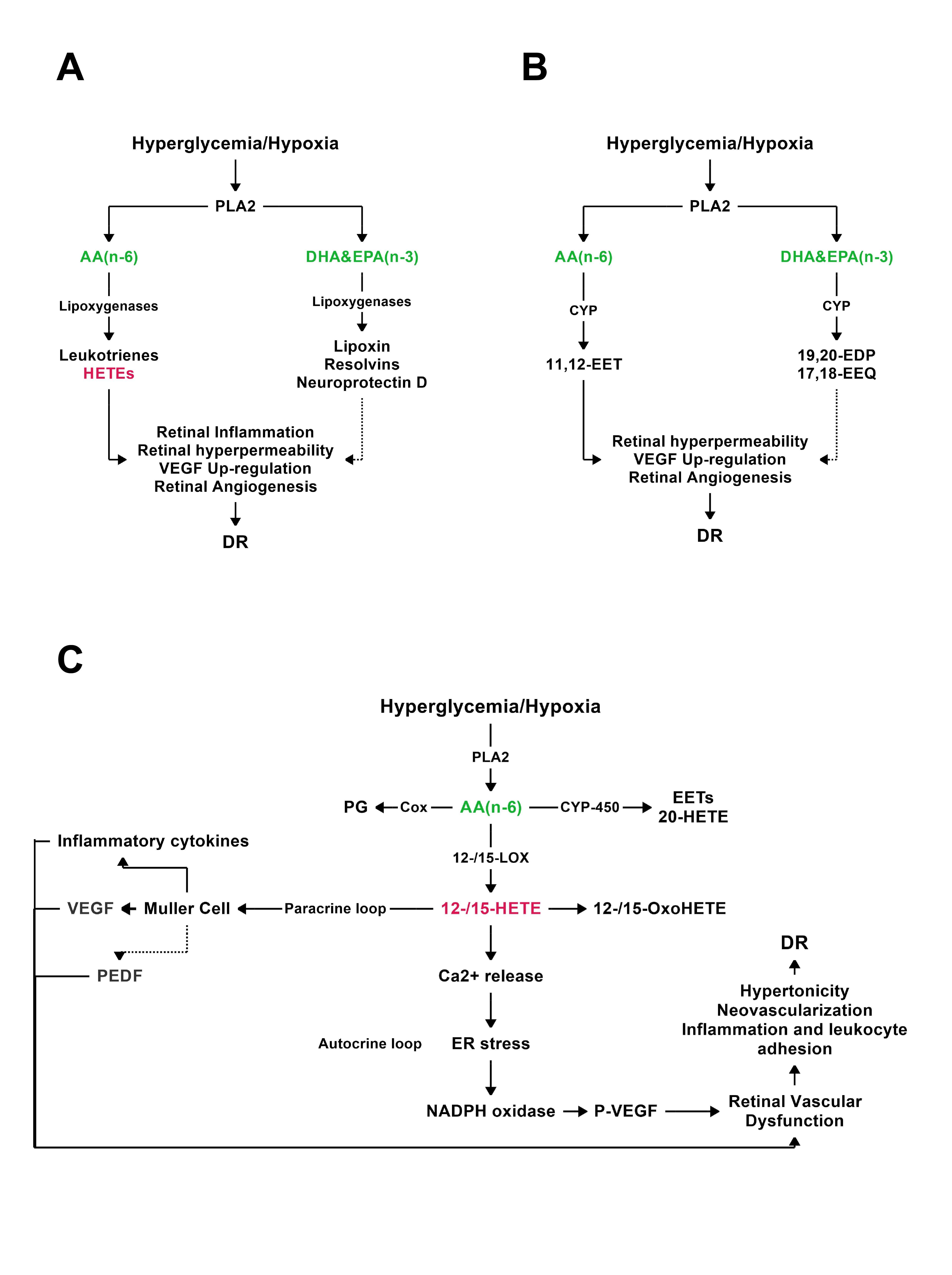


**Figure S9.** Ploy unsaturated fatty acid-derived cascade events involved in the pathogenesis of DR. Red text represents significant increase; green text represents significant decrease; the dashed line represents inhibition.

# *Abbreviations: DR: diabetic retinopathy; AA: arachidonic acid; DHA: docosahexaenoic acid; EPA: eicosapentanoic acid; PLA2: phospholipase A2; Cox: Cyclooxygenases; CYP-450: Cytochrome P450; LOX: Lipoxygenase**; HETE: hydroxyeicosatetraenoic acid**; VEGF: vascular endothelial growth factor; PEDF: pigment epithelium-derived factor; ER: endoplasmic reticulum.*
